# OP03 The Distribution Of Quality-Adjusted Life Expectancy By Level Of Socioeconomic Disadvantage And Remoteness Area For The Australian Population

**DOI:** 10.1017/S0266462325100627

**Published:** 2025-12-29

**Authors:** Sheridan Rodda, Jedidiah Morton, Melanie Lloyd, Richard Norman, Zanfina Ademi

## Abstract

**Introduction:**

Equity-informed economic evaluations require the baseline distribution of health across equity subgroups upon which the equity impact of interventions is evaluated. The distribution of health status, measured as quality-adjusted life expectancy (QALE), by socioeconomic status (SES) is unknown for Australia. We aimed to estimate QALE across SES groups, stratified by sex and year of age, for the Australian population.

**Methods:**

SES was measured with the Socio-Economic Indexes for Areas Index of Relative Socio-Economic Disadvantage, from quintile one (Q1) most socioeconomically disadvantaged, to quintile five (Q5) least disadvantaged, and remoteness categories from the Australian Statistical Geography Standard: major cities, inner regional, and outer regional to very remote. Life expectancy (LE) was estimated from 2022 Australian Bureau of Statistics mortality data. Mean short form (SF-6D) utility by age, sex, and SES was estimated from the Household, Income and Labour Dynamics in Australia Survey (2022) using linear regression. Person-years were multiplied by utility to determine the QALE for each sex-SES group.

**Results:**

At birth, LE for all individuals in Q1 was 78.7 years, compared with 86.3 years for those in Q5. Incorporating health-related quality of life amplified disparities, with those in Q1 experiencing a QALE of 43.9 (95% confidence interval [CI]: 42.6, 45.2) years, compared with 55.6 (95% CI: 54.1, 57.1) years for Q5, a 27 percent relative difference. There were modest disparities by remoteness. Individuals in major cities had a LE of 83.1 years and a QALE of 50.8 (95% CI: 49.8, 51.7) years, while those in outer regional to remote areas had a LE of 80.5 years and a QALE of 47.0 (95% CI: 44.2, 49.7) years.

**Conclusions:**

There is clear disparity in QALE by SES in Australia, whereby both the quantity and quality of life decrease with increasing socioeconomic disadvantage and geographical remoteness. These findings highlight the need for targeted interventions to address health inequalities. These detailed QALE estimates can be applied to future equity-informed economic evaluations.

